# *In Vitro* Activity of Tedizolid, Dalbavancin, and Ceftobiprole Against *Clostridium difficile*

**DOI:** 10.3389/fmicb.2018.01256

**Published:** 2018-06-11

**Authors:** Dana Binyamin, Orna Nitzan, Maya Azrad, Zohar Hamo, Omry Koren, Avi Peretz

**Affiliations:** ^1^Clinical Microbiology Laboratory, Baruch Padeh Medical Center, Poriya, Azrieli Faculty of Medicine, Bar Ilan University, Galilee, Israel; ^2^The Azrieli Faculty of Medicine, Bar Ilan University, Galilee, Israel; ^3^Unit of Infectious Diseases, Baruch Padeh Medical Center, Poriya, Azrieli Faculty of Medicine, Bar Ilan University, Galilee, Israel

**Keywords:** *Clostridium difficile*, vancomycin, tedizolid, dalbavancin, ceftobiprole, ceftriaxone

## Abstract

**Background:** *Clostridium difficile* (*C. difficile*) is a major nosocomial pathogen that colonizes in the human gut. Recently, the U.S. FDA approved three new antimicrobial agents against gram-positive bacteria: Tedizolid, Dalbavancin, and Ceftobiprole. The efficacy of these antibiotics for treatment of *C. difficile* infection has not been thoroughly examined. The current study aimed to examine the *in vitro* activity of these antibiotics against *C. difficile*. In addition, to compare between Dalbavancin and Ceftobiprole to antibiotics from the same class: Vancomycin and Ceftriaxone, respectively.

**Methods:** Eighty-four *C. difficile* isolates were tested for susceptibility to Tedizolid, Dalbavancin, Ceftobiprole, Vancomycin, and Ceftriaxone by Etest technique in order to determine the minimum inhibitory concentration (MIC).

**Results:** Upon comparison of the novel antibiotic agents, Dalbavancin demonstrated the lowest MIC values and ceftobiprole the highest at MIC_50_ (0.016, 0.38, and 1.5 μg/mL, for Dalbavancin, Tedizolid, and Ceftobiprole, respectively) and MIC_90_ (0.03, 0.78, and 3.17 μg/mL, respectively). Dalbavancin demonstrated significantly lower MIC_50_ and MIC_90_ values compared to Vancomycin (0.016 vs. 0.38 and 0.03 vs. 3.5, respectively) (*p* < 0.001) and ceftobiprole had significantly lower MIC values compare to ceftriaxone (1.5 vs. 32 and 3.17 vs. 28.8, respectively) (*p* < 0.001).

**Conclusion:** Dalbavancin and Tedizolid may play a role as potential therapeutic agents for treatment of *C. difficile* infection. Examination of antibiotic effect on the intestinal microbiome and clinical trials are needed for more accurate results.

## Introduction

*Clostridium difficile* is a gram-positive rod, an obligate anaerobe, spore forming, toxin-producing bacterium that colonizes the human gut (Hall and O’Toole, 1935). The incidence of *C. difficile* infection (CDI) has increased markedly worldwide since 2000 (Kelly and LaMont, 2008). In Israel, the incidence of *C. difficile* infection in 2016 was 2761 cases per 100,000 patients (Israeli National Center for Infection Control, unpublished data). Disease symptoms include diarrhea and abdominal pain, fever, and increased levels of blood lymphocytes. In severe cases, pseudomembranous colitis, toxic megacolon, or colonic perforation may occur, with high mortality rates (Johanesen et al., 2015). The highly resistant spores can survive on surfaces for long periods, and thus be easily transferred from person to person. This occurs mainly in hospitals and long-term care facilities; therefore CDI is a nosocomial infection and has important clinical and financial implications for hospitals (Paredes-Sabja et al., 2014).

The major risk factor for CDI is antibiotic administration, which disrupts the normal intestinal microbiota, leading to spore germination and proliferation of *C. difficile.* Although nearly all antimicrobial classes have been associated with CDI, clindamycin, third-generation cephalosporins, fluoroquinolones, and penicillins are most commonly associated with this disease (Owens et al., 2008).

Several treatment strategies exist; in some cases, cessation of antibiotics that induced CDI is sufficient for a cure. However, the majority of patients are treated with antibiotics such as metronidazole and Vancomycin (Davies et al., 2011). In severe and recurrent cases, fecal microbiota transplantation, a treatment that restores the normal fecal microbiota, is efficacious (Youngster et al., 2014).

Recently, the U.S. FDA approved three new antimicrobial agents against gram-positive bacteria: Tedizolid, Dalbavancin, and Ceftobiprole. Tedizolid is an oxazolidinone derivate with higher potency than Linezolid, requiring a lower dose for effective result (Im et al., 2011; Prokocimer et al., 2013; Sahm et al., 2015). Tedizolid is highly active against gram-positive bacteria, and can be used for the treatment of acute bacterial skin and skin structure infections (Ferrández et al., 2017). This antibiotic binds to the 50S subunit of the bacterial ribosome and inhibits protein synthesis (Shaw et al., 2008).

Dalbavancin is a bactericidal lipoglycopeptide antibiotic that inhibits cell wall synthesis. Similar to Vancomycin, it belongs to the glycopeptide antibiotic group and is an efficient treatment for infections caused by Vancomycin-resistant strains (Tatarkiewicz et al., 2016). *In vitro* Dalbavancin activity against various gram-positive species, including *C. difficile*, was more potent compared to Vancomycin (Goldstein et al., 2003; Huband et al., 2016; Tatarkiewicz et al., 2016).

Ceftobiprole is a new generation of cephalosporin with broad-spectrum activity against gram-positive and gram-negative bacteria (Ednie et al., 2007). Ceftobiprole is a β-lactam antibacterial with bactericidal activity (Murthy and Schmitt-Hoffmann, 2008).

The current study aimed to examine the *in vitro* activity of Tedizolid, Dalbavancin, and Ceftobiprole against *C. difficile* by detecting the minimum inhibitory concentration (MIC). In addition, we also compared Dalbavancin and Ceftobiprole to Vancomycin and Ceftriaxone, respectively, antibiotics from the same class.

## Materials and Methods

### Study Population

Patients diagnosed with *C. difficile* infection at the Baruch Padeh Medical Center, Poriya in northern Israel, were enrolled in the study from January 2015 to May 2017. The identification of CDI was performed by stool examination for toxigenic *C. difficile* at the Clinical Microbiology Laboratory by Xpert^®^ *C. difficile* Assay (Cepheid, Solna, Sweden), performed on Cepheid GeneXpert^®^ Systems. This is a qualitative *in vitro* real-time PCR for the rapid identification of *C. difficile*. The study was approved by the Poriya Baruch Padeh Medical Center Helsinki Committee without the need for patients to sign an informed consent form because the study deals with microbial isolates and the results of the study do not affect the patients.

### Bacteria Isolation and Identification

For this purpose, 0.5 mL of liquid feces was suspended in 4.5 mL physiological solution. Fifty μL of the suspension were inoculated on a selective CHRomagar medium; chromID^TM^ *C. difficile* (bioMérieux, France) and then incubated at 37°C in anaerobic conditions (GasPakTM EZ, BD, United States) for 48 h. *C. difficile* colonies appear as asymmetric and black-colored colonies. Final identification was done by matrix-assisted laser desorption ionization-time of flight mass spectrometry (MALDI-TOF MS)-based technology using the Bruker Biotyper system (Bruker, United States).

### Antibiotic Susceptibility Tests

Antibiotic susceptibility tests were performed by the Etest technique in order to determine the minimum inhibitory concentration (MIC), which is the lowest antibiotic concentration that inhibits bacterial growth. To this end, bacteria colonies were suspended in saline, generating turbidity of 0.5 McFarland. The suspensions were seeded on Mueller Hinton + 5% Sheep Blood agar plate (HyLaboratories, Rehovot, Israel). Then, a gradient Etest strip (Liofilchem, Italy) for each antibiotic was added to each plate and incubated at 37°C in anaerobic conditions for 48 h. After incubation, the susceptibility breakpoint was determined as the lowest concentration at which no bacterial growth was detected. Additionally, MIC_90_ and MIC_50_ were calculated as the MICs at which 90%/50% of the isolates tested are inhibited. Etest procedures were done for Tedizolid, Dalbavancin, and Ceftobiprole, as well as for Vancomycin and Ceftriaxone for comparison to Dalbavancin and Ceftobiprole, respectively.

### Statistical Analysis

Differences between the MIC_50_ or MIC_90_ of Tedizolid, Dalbavancin, and Ceftobiprole were analyzed by one-way analysis of variance (ANOVA), with Benferroni’s *post hoc* test.

Differences between the MIC_50_ or MIC_90_ of Ceftobiprole and ceftriaxone as well as the differences between the MIC_50_ or MIC_90_ of Dalbavancin and Vancomycin, were analyzed by Wilcoxon matched-pairs signed rank test.

Statistical significance was defined by *p* < 0.05.

## Results

Eighty-four *C. difficile* isolates were tested for susceptibility to Tedizolid, Dalbavancin, and Ceftobiprole. MIC_50_ and MIC_90_ results are presented in **Table 1**. The MIC_50_ and MIC_90_ results of each antibiotic were different from the other antibiotic agents (*p* < 0.001). Dalbavancin demonstrated low MIC results compared to Tedizolid and Ceftobiprole, for MIC_50_ (0.016, 0.38, and 1.5 μg/mL, respectively) and MIC_90_ (0.03, 0.78, and 3.17 μg/mL, respectively). Ceftobiprole was the antibiotic with the highest MIC values compared to the two other antibiotics.

**Table 1:** Susceptibility test (MICs) of *C. difficile* isolates.

Antibiotic		Tedizolid	Dalbavancin	Ceftobiprole	*p*-Value
**MIC (μg/mL)**	**Range**	0.032–32	0.002–0.250	0.016–32	<0.001
	**MIC_50_**	0.38	0.016	1.5	
	**MIC_90_**	0.78	0.03	3.17	

**FIGURE 1:**
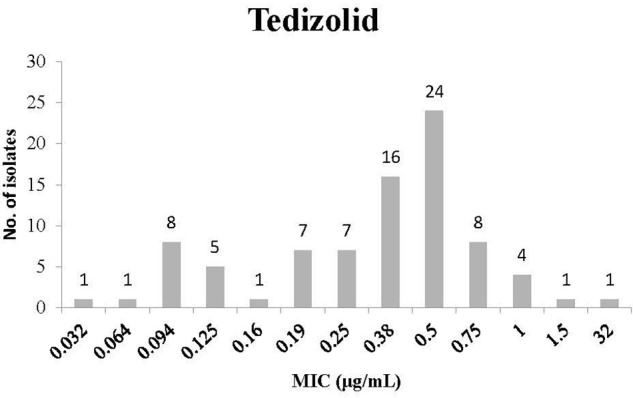
Minimum inhibitory concentration (MIC) distribution of *Clostridium difficile* isolates to Tedizolid.

The distribution of MIC for Tedizolid is presented in **Figure 1**. Most isolates had an MIC of 0.5 μg/mL (24 isolates) or 0.38 μg/mL (16 isolates). In addition, some of the isolates had low MIC values (for instance 8 isolates with 0.094 μg/mL and 5 isolates with 0.125 μg/mL). However, other isolates had higher MIC values (for instance 1 isolate with an MIC of 32 μg/mL and 8 isolates with an MIC of 0.75 μg/mL).

**FIGURE 2:**
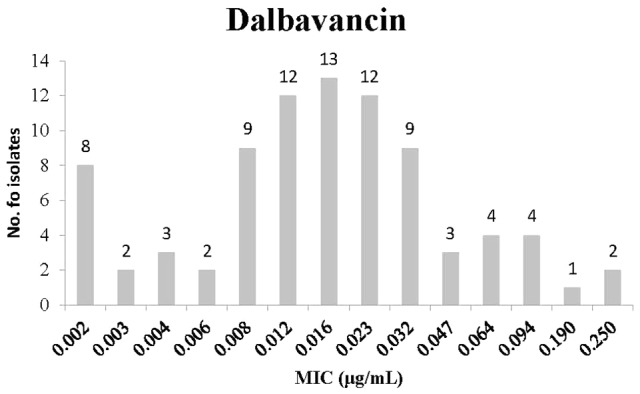
Minimum inhibitory concentration distribution of *C. difficile* isolates to Dalbavancin.

**Figure 2** presents the MIC distribution for Dalbavancin. Overall, Dalbavancin susceptibility results demonstrated low MIC values. Most isolates had an MIC of 0.016 μg/mL (13 isolates) and 0.012 or 0.023 μg/mL (12 isolates).

**FIGURE 3:**
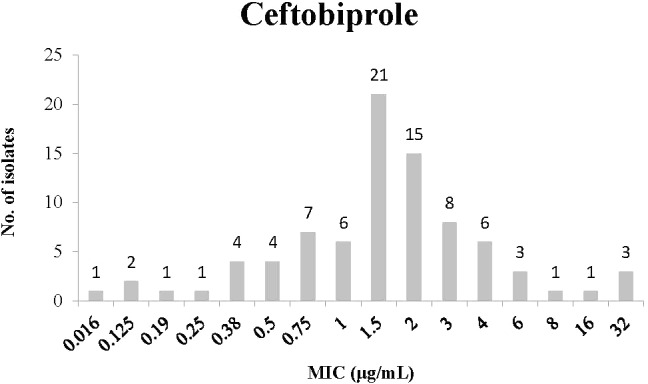
Minimum inhibitory concentration distribution of *C. difficile* isolates to Ceftobiprole.

Minimum inhibitory concentration distribution for Ceftobiprole is presented in **Figure 3**. Most isolates had an MIC of 1.5 μg/mL (21 isolates) or 2 μg/mL (15 isolates). Many isolates had high MIC values; for instance 8 isolates with MIC of 3 μg/mL, 6 with 4 μg/mL, and 3 with 32 μg/mL.

We compared the susceptibility of antibiotics from the same antibiotic group (**Table 2**). In a comparison of the MIC_50_ and MIC_90_ between Dalbavancin and Vancomycin, Dalbavancin showed significantly lower MIC values (0.016 vs. 0.38 and 0.03 vs. 3.5, respectively, *p* < 0.001).

**Table 2:** Comparison between susceptibility tests (MICs) of antibiotics in the same antibiotic group.

Antibiotic		Dalbavancin	Vancomycin	*p*-Value	Ceftobiprole	Ceftriaxone	*p-*Value
**MIC (μg/mL)**	**Range**	0.002–0.250	0.016–256	<0.001	0.016–32	0.38–32	<0.001
	**MIC_50_**	0.016	0.38		1.5	32	
	**MIC_90_**	0.03	3.5		3.17	28.8	

In comparison of the MIC_50_ and MIC_90_ between Ceftobiprole and Ceftriaxone, Ceftobiprole had significantly lower MIC values (1.5 vs. 32 and 3.17 vs. 28.8, respectively, *p* < 0.001).

## Discussion

Antibiotic administration is the main risk factor for CDI, even though treatment of the disease includes antibiotic therapy. Despite the efficacy of the conventional antibiotics for treating the disease, treatment failure, recurrence, and antibiotic resistance are reported (Adler et al., 2015; Freeman et al., 2015). Therefore, it is important to identify new antibiotics that may potentially be used to treat CDI.

The current study examined the MIC of three recently approved antibiotics: Tedizolid, Dalbavancin, and Ceftobiprole. These antibiotics are affective against gram-positive bacteria such as methicillin resistant *Staphylococcus aureus* and Vancomycin resistant *Enterococci* (Ednie et al., 2007; Im et al., 2011; Huband et al., 2016). However, antibiotic efficacy against *C. difficile* has not been thoroughly examined.

Of all three antibiotics, Dalbavancin was found to be the most effective antibiotic with high prevalence of isolates with low MIC values. In comparison to Vancomycin, another lipoglycopeptide antibiotic, Dalbavancin, had significantly lower MIC values for *C. difficile*. These results are different from those found in a previous study, where higher MIC levels for Dalbavancin and lower MIC levels for Vancomycin were demonstrated, compared with our findings, but similar to what we found – MIC values for Dalbavancin were lower than those of Vancomycin (Goldstein et al., 2003). Vancomycin is considered first line treatment of moderate to severe CDI (Davies et al., 2011). Different studies reported resistance rates of 7.4–47% to Vancomycin, treatment failure in 14.2% of cases, and recurrence rate of 24.0% (Adler et al., 2015; Freeman et al., 2015; Tkhawkho et al., 2017). Due to the presence of Vancomycin-resistant strains and recurrence cases, Dalbavancin might be used as treatment for CDI. Further research is required, mainly clinical trials and examination of antibiotic effect on the intestinal microbiome.

Ceftobiprole had the highest MIC values for *C. difficile* compared to the other examined antibiotics, although these MIC values were lower than those found in another study (Ednie et al., 2007). In comparison to another broad spectrum cephalosporin, ceftriaxone, *C. difficile* isolates had significantly lower MIC values. Cephalosporins have poor *in vitro* activity against *C. difficile* (Wilcox et al., 2016). Some cephalosporins even promote *C. difficile* spore germination, proliferation, and toxin production (Wilcox et al., 2016). However, a study in a mouse model that tested Ceftobiprole’s effect on *C. difficile* found that Ceftobiprole does not promote the growth of *C. difficile* or toxin production, in contrast to Ceftazidime, Cefotaxime, and Ceftriaxone (Nerandzic and Donskey, 2011). Another study that investigated the effect of Ceftobiprole administration on normal intestinal microbiota found that it had no significant ecological impact on the human intestinal microbiota (Bäckström et al., 2010). Consequently, Ceftobiprole can be used for CDI treatment and when administered for other indications may be associated with a reduced risk for CDI compared with other cephalosporins.

We also found a high prevalence of isolates with low MIC values to Tedizolid. This antibiotic was shown to have high activity against gram-positive bacteria, mainly skin pathogens, with low MIC rates (Bensaci and Sahm, 2017; Ferrández et al., 2017). Ours is the first study that examines the effect of Tedizolid on *C. difficile*. In a clinical trial, fewer patients who had received Tedizolid suffered from gastrointestinal side effects (16%) than those who received Linezolid (23%) (Shorr et al., 2015). Further research is warranted to determine whether Tedizolid is effective against *C. difficile*.

## Conclusion

The activity of three novel antibiotics – Tedizolid, Dalbavancin, and Ceftobiprole – against *C. difficile* was examined. Dalbavancin and Tedizolid may be potential therapeutic agents for the treatment of CDI. Clinical trials are needed to confirm the laboratory experiments, as well as studies that examine the effect of these antibiotics on the intestinal microbiome to ensure they are not a risk factor for CDI.

## Author Contributions

DB, ON, OK, and AP designed the study and interpreted the data. AP, MA, and ON drafted the manuscript. DB, ZH, MA, and AP performed laboratory work. All authors read and approved the final manuscript.

## Conflict of Interest Statement

The authors declare that the research was conducted in the absence of any commercial or financial relationships that could be construed as a potential conflict of interest.
